# Phosphoproteomic Analysis of the Amygdala Response to Adolescent Glucocorticoid Exposure Reveals G-Protein Coupled Receptor Kinase 2 as a Target for Reducing Motivation for Alcohol

**DOI:** 10.3390/proteomes6040041

**Published:** 2018-10-12

**Authors:** Megan L. Bertholomey, Kathryn Stone, TuKiet T. Lam, Seojin Bang, Wei Wu, Angus C. Nairn, Jane R. Taylor, Mary M. Torregrossa

**Affiliations:** 1 Department of Psychiatry, University of Pittsburgh, 450 Technology Dr. Bridgeside Point II, Suite 223, Pittsburgh, PA 15219, USA; bertholomeym@upmc.edu; 2 Keck Foundation Biotechnology Resource Laboratory, Yale University, New Haven, CT 06536, USA; gstonecreek@gmail.com (K.S.); tukiet.lam@yale.edu (T.T.L.); 3 Department of Molecular Biophysics and Biochemistry, Yale University, New Haven, CT 06508, USA; 4 Computational Biology Department, Carnegie Mellon University, Pittsburgh, PA 15213, USA; seojinb@andrew.cmu.edu (S.B.); weiwu2@cs.cmu.edu (W.W.); 5 Department of Psychiatry, Yale University, New Haven, CT 06508, USA; angus.nairn@yale.edu (A.C.N.); jane.taylor@yale.edu (J.R.T.); 6 Department of Psychology, Yale University, New Haven, CT 06508, USA

**Keywords:** adolescence, corticosterone, proteomics, yohimbine, progressive ratio, reinstatement, ethanol

## Abstract

Early life stress is associated with risk for developing alcohol use disorders (AUDs) in adulthood. Though the neurobiological mechanisms underlying this vulnerability are not well understood, evidence suggests that aberrant glucocorticoid and noradrenergic system functioning play a role. The present study investigated the long-term consequences of chronic exposure to elevated glucocorticoids during adolescence on the risk of increased alcohol-motivated behavior, and on amygdalar function in adulthood. A discovery-based analysis of the amygdalar phosphoproteome using mass spectrometry was employed, to identify changes in function. Adolescent corticosterone (CORT) exposure increased alcohol, but not sucrose, self-administration, and enhanced stress-induced reinstatement with yohimbine in adulthood. Phosphoproteomic analysis indicated that the amygdala phosphoproteome was significantly altered by adolescent CORT exposure, generating a list of potential novel mechanisms involved in the risk of alcohol drinking. In particular, increased phosphorylation at serines 296–299 on the α_2A_ adrenergic receptor (α_2A_AR), mediated by the G-protein coupled receptor kinase 2 (GRK2), was evident after adolescent CORT exposure. We found that intra-amygdala infusion of a peptidergic GRK2 inhibitor reduced alcohol seeking, as measured by progressive ratio and stress reinstatement tests, and induced by the α_2A_AR antagonist yohimbine. These results suggest that GRK2 represents a novel target for treating stress-induced motivation for alcohol which may counteract alterations in brain function induced by adolescent stress exposure.

## 1. Introduction

Chronic stress is an environmental factor known to increase the risk for psychiatric disorders, including alcohol use disorders. Importantly, chronic stress during critical developmental periods can have long-lasting effects on alcoholism risk [1,2,3,4,5]. Specifically, exposure to multiple adverse events in childhood, including adolescence, is associated with greater lifetime incidence and earlier onset of alcohol dependence [6,7]. Adolescence may be a period of particular vulnerability because of the ongoing development of brain circuits responsive to glucocorticoids during that time. Indeed, we have found that chronic exposure to the glucocorticoid stress hormone corticosterone (CORT) during adolescence, using an established procedure that produces a depression-like syndrome in adults [8,9], increases impulsivity on the delay-discounting test of impulsive choice, indicating that adolescent CORT has long-term effects on behavior [10]. Moreover, impulsivity on delay discounting tasks is frequently associated with presence of alcohol use disorders, and it is a possible risk factor for alcoholism [11].

Preclinical studies suggest that stress hormone exposure in adolescence may influence motivation for ethanol in adulthood, including reports demonstrating that post-weaning social isolation stress for either 42 [12,13] or 90 [14] days, which includes but is not limited to the adolescent period, can increase subsequent operant ethanol self-administration. Moreover, we recently reported that adolescent corticosterone exposure from postnatal day (PND) 30–50 can increase a variety of alcohol-motivated behaviors in male and female rats [15]. 

In Experiment 1 of the current study, we demonstrate that under certain training conditions, male rats exposed to CORT in adolescence demonstrate an increased motivation for alcohol, as evidenced by increased operant alcohol self-administration and yohimbine-induced reinstatement of alcohol-seeking behavior in adulthood, whereas responding for sucrose was unchanged. We then used a discovery-based phosphoproteomics approach to determine what signaling systems were persistently altered in the amygdala of rats exposed to CORT in adolescence, which might interact with the ethanol self-administration experience. The proteomics analysis revealed several persistent changes in protein phosphorylation based on adolescent experience that may represent potential mechanisms underlying increased motivation for alcohol, including increased phosphorylation of the α_2A_ adrenergic receptor (α_2A_AR) by CORT. Consequently, Experiment 2 sought to determine the effects of direct manipulation of α_2A_AR function on adult alcohol-motivated behavior in adolescent CORT-exposed male rats.

## 2. Materials and Methods

### 2.1. Subjects

Male Sprague Dawley rats aged 24–27 days upon delivery to the animal facility were used in all experiments. Experiment 1 was conducted at Yale University in the Connecticut Mental Health Center, and Experiment 2 was conducted at the University of Pittsburgh. Rats were obtained from Charles River (Kingston, NY, USA) at Yale and from Harlan/Envigo (Frederick, MD, USA) at the University of Pittsburgh. We used different vendors to minimize animal shipping time to both facilities. In addition, at Yale, rats were housed in shoebox cages with water bottles on standard racks, while at the University of Pittsburgh, rats were housed in individually ventilated caging (IVC) with an automated watering system. All other housing and procedural parameters were the same between the two universities, unless otherwise noted. In addition, all procedures were conducted in accordance with the National Institutes of Health *Guide for the Care and Use of Laboratory Animals* and were approved by each institution’s Institutional Animal Care and Use Committee. Rats acclimated to the facility for 3–5 days before CORT exposure began on postnatal day (PND) 30. Rats were pair-housed and maintained on a 12:12 hour light-dark cycle in a temperature- and humidity-controlled environment. The rats were given ad libitum access to food and water except during periods of food restriction, as described below. 

### 2.2. Drugs

Corticosterone hemisuccinate (CORT; 4-pregnen-11β,21-diol-3,20-dione21-hemisuccinate, Steraloids, Newport, RI, USA) was prepared fresh every three days. CORT was dissolved in tap water and stirred overnight at a pH of 10–11 and neutralized to a pH of 7.0–7.4 prior to use. Ethanol (EtOH; Decon Labs, King of Prussia, PA, USA) and saccharin (Acros Organics, Pittsburgh, PA, USA) were diluted in tap water to concentrations of 10% (*v*/*v*) and 0.1% (*w*/*v*), respectively, to make the sweetened EtOH solution. Yohimbine (Sigma, St. Louis, MO, USA) was dissolved in double distilled water to a concentration of 1.25 mg/mL. GRK2i (GRK2 inhibitory polypeptide; Tocris, Minneapolis, MN, USA) was dissolved in saline to a concentration of 2 mM.

### 2.3. Chronic Corticosterone Exposure

From postnatal day (PND) 30–50, a period which spans the majority of adolescence in rodents [1], rats received access to a bottle containing either water or a solution of CORT as their sole source of fluid (note: the automated watering system was disabled in Experiment 2). For the first 14 days of exposure, rats received a concentration of 50 μg/mL CORT, which was then reduced to 25 μg/mL and finally to 12.5 μg/mL for three days each. During the exposure period, CORT- and water (H_2_O)-containing bottles were weighed daily, and rats were weighed every other day. Following cessation of CORT exposure, all rats were returned to normal tap water. Behavioral testing began following a 10-day washout period to allow for the re-establishment of endogenous hypothalamic-pituitary-adrenal (HPA) axis functioning [9] and for the rats to age into adulthood. The CORT exposure procedure was the same as that previously described and that has been reported to produce circulating CORT levels of greater than 800 ng/mL [9,16]. 

### 2.4. Operant Self-Administration 

All rats remained CORT-free during behavioral testing. To facilitate acquisition of self-administration, rats were mildly food restricted during training. In Experiment 1, rats were maintained on this restriction, while in Experiment 2, the restriction was gradually eased such that rats were fed ad libitum by the end of training. Self-administration sessions were conducted in standard operant chambers (MedAssociates, St. Albans, VT, USA) housed in sound-attenuating cubicles. Rats were trained to respond for 10 s presentation of the reinforcer paired with a light + tone cue on a fixed ratio (FR)1 schedule of reinforcement. In both experiments, rats were trained to self-administer a solution of 10% (*v*/*v*) EtOH + 0.1% (*w*/*v*) saccharin, and in Experiment 1, a control group of rats were trained to self-administer a solution of 20% (*w*/*v*) sucrose. Sucrose was used for comparison as it contains calories similar to ethanol, and has a sweet taste, similar to saccharin. In Experiment 1, rats received 20 one-hour self-administration sessions during the light cycle. In Experiment 2, sessions were conducted in the dark cycle and were initially 30 min in length, and they were subsequently extended to 60 min to match the duration of Experiment 1. Rats received a total of 21 self-administration sessions prior to surgery, and an additional 10 sessions following surgery to re-establish baseline responding.

### 2.5. Experiment 1. Analysis of Adolescent CORT Effects on Adult Ethanol Self-Administration and the Amygdala Phosphoproteome

#### Quantitative Label-Free Phosphoproteomics

Fourteen to 16 days after the last day of self-administration, rats from Experiment 1 were euthanized by focused microwave irradiation, in order to analyze the amygdala phosphoproteome using high resolution tandem mass spectrometry. Focused microwave irradiation is known to maintain the post-translational modification state of proteins during the post-mortem period [17]. Rats were lightly anesthetized with isoflurane prior to euthanasia, to reduce the influence of acute stress on protein phosphorylation. After euthanasia, the amygdala was dissected and homogenized by sonication in a buffer containing urea (ThermoFisher, 8 M), ammonium bicarbonate (ThermoFisher, 0.4 M), and protease (Pierce, at 1% of lysis buffer) and phosphatase inhibitor cocktails (Pierce, at 2.5% of lysis buffer). Samples from two rats in each experimental group were randomly pooled to create a total of four biological samples per group. Pooled samples were then analyzed by the Yale/NIDA Neuroproteomics Center as previously described [18]. Briefly, 20 µL of 45 mM dithiothreitol (DTT) was added to each sample and incubated at 37 °C for 20 min to reduce Cys residues. Samples were cooled and 20 µL of 100 mM iodoacetamide (IAM) was added to each sample and incubated at room temperature in the dark for 20 min for alkylation of the reactive free sulfhydro of the reduced Cys. Dual enzymatic digestion was carried out by adding 600 µL of dH_2_O and 30 µL of 1 mg/mL Lys C followed by incubation at 37 °C for 4 h, with subsequent digestion by incubation with 30 µL of 1 mg/mL trypsin overnight at 37 °C. Samples were macrospin desalted and dried by a Speedvac. Pellets were dissolved in 50 µL of a solution containing 0.5% trifluoroacetic acid (TFA) and 50% acetonitrile. Samples were then subjected to titanium dioxide (TiO_2_) phosphopeptide enrichment using TopTips (Glygen, Columiba, MD, USA). A three-step conditioning of the TopTip was utilized with 1 min at 2000 rpm on a bench top centrifuge (ThermoFisher) for each step. First, the TopTip was washed with 2 × 60 µL 100% acetonitrile, then with 2 × 60 µL 0.2 M sodium phosphate (pH 7.0), and finally with 2 × 60 µL 0.5% TFA in a 50% acetonitrile solution. The acidified digest supernatants were loaded into the TopTip, and bound phosphopeptides were washed with 2 × 40 µL of a buffer containing 0.5% TFA in 50% acetonitrile, spun at 1000 rpm for 1 min, and then at 3000 rpm for 2 min. Phosphopeptides were eluted from each TopTip by three aliquots of 30 µL of 28% high purity ammonium hydroxide (ThermoFisher). The eluted fraction was dried and re-dried with 2 × 30 µL water by speedvac. Enriched fractions were dissolved in 10 µL of 70% formic acid and 30 µL of 50 mM sodium phosphate. Peptide concentrations were determined by NanoDrop to load 0.3 µg/5 µL of each sample.

For LC/MS-MS, 5 µL of each sample was injected onto a LTQ Orbitrap XL LC-MS/MS system. Peptide separation was performed on the nanoACQUITY™ ultra-high pressure liquid chromatography (UPLC™) system (Waters, Milford, MA), using a Waters Symmetry^®^ C18 180 µm × 20 mm trap column and a 1.7 µm, 75 µm × 250 mm nanoACQUITY™ UPLC™ column (35 °C). Trapping was done at 15 µL/min, with 99% Buffer A (0.1% formic acid in water) for 1 min. Peptide separation was performed over 120 min at a flow rate of 300 nL/min beginning with 95% Buffer A and 5% Buffer B (0.075% formic acid in acetonitrile) to 40% B from 1–9 min, to 85% B from 9–91 min, held at 85% B from 91–95 min, then returned to 5% B from 95–96 min. Two washes were made between each sample run to ensure no carryover (1. 100% acetonitrile, 2. Buffer A). The LC was in-line with an LTQ-Orbitrap XL mass spectrometer. MS was acquired in the Orbitrap using one microscan, and a maximum injection time of 900 msec followed by 3–6 data-dependent MS/MS acquisitions in the ion trap (with precursor ion threshold of >3000). The total cycle time for both MS and MS/MS fragmentation by collision induced dissociation (CID) were first isolated with a 2 Da window, followed by normalized collision energy of 35%. Dynamic exclusion was activated where former target ions were excluded for 30 s. Three technical replicates were injected for each sample and all samples and replicates were randomized across the entire run time. 

### 2.6. Experiment 2. Determining the Role of GRK2 in Regulating Ethanol-Motivated Behaviors

The proteomics analysis revealed several changes in protein phosphorylation of potential interest for mediating the increase in motivation to respond for alcohol in adolescent CORT-exposed rats. One of particular relevance was the increase in phosphorylation of the α_2A_ adrenergic receptor (α_2A_R) on serines (S) 296–299 in the amygdala of CORT-exposed animals relative to controls. Phosphorylation of these four neighboring residues is mediated by the G-protein coupled receptor kinase 2 (GRK2, also known as the β-adrenergic receptor kinase-βARK) [19]. Therefore, we sought to determine whether inhibition of GRK2 in the amygdala could prevent or reduce yohimbine-induced increases in alcohol-motivated behavior that were measured by progressive ratio responding for alcohol, and reinstatement of alcohol seeking. 

#### 2.6.1. Surgery 

Rats were anesthetized with a combination of 87.5 mg/kg ketamine and 5 mg/kg xylazine, and were injected with 5 mg/kg of Rimadyl (NSAID analgesic) and 5 mL of lactated Ringers’ solution prior to surgery. Rats were implanted bilaterally with intracranial guide cannulae (28 gauge, Plastics One) aimed 1 mm above the basolateral amygdala using the following coordinates: from bregma: AP −3.0 mm; ML ±5.3 mm; DV −7.9 mm. Cannulae were anchored with three stainless steel screws and dental cement, and obturators were placed within the cannulae to maintain patency. Rats were monitored for seven days post-surgery and then they resumed EtOH self-administration. 

#### 2.6.2. Progressive Ratio (PR) 

The PR schedule began with a ratio of 1 that increased by 2 within each step, then it increased by 1 every four steps (e.g., 1, 3, 5, 7; 10, 13, 16, 19; 23, 27, 31, 35; etc.). Rats were first given three baseline PR sessions, the last of which included a sham injection to acclimate the rats to the infusion/injection procedure. On test days, rats were infused with 1 nmol/side of GRK2i (vs vehicle (saline), between-subjects) 10 min prior to an intraperitoneal injection of 1.25 mg/kg yohimbine (vs. vehicle (H_2_O), within-subjects] given 10 min prior to each of two PR sessions. Yohimbine and vehicle PR sessions were given in a counterbalanced manner and were separated by a non-injection PR session.

#### 2.6.3. Extinction/Yohimbine-Induced Reinstatement of EtOH Seeking

Following self-administration (Experiment 1) or PR testing (Experiment 2), rats underwent at least five days of extinction (no more than 10 days) until meeting the extinction criterion (≤20 active lever presses over two consecutive days). In Experiment 1, rats were tested for stress-induced reinstatement of EtOH seeking following challenge with yohimbine and vehicle in two separate test days [15,20,21]. Similarly, in Experiment 2, rats were tested in two reinstatement sessions (yohimbine and vehicle) using the same infusion/injection parameters described above for PR testing. Assignment to GRK2i and vehicle groups was counterbalanced such that some rats received the same infusion for both PR and reinstatement (e.g., GRK2i, GRK2i or VEH, VEH) while others received the opposite infusion (e.g., GRK2i, VEH or VEH, GRK2i).

### 2.7. Statistical Analysis

#### 2.7.1. Behavioral Data

Behavioral data were analyzed by multi-factor ANOVA, including repeated measures where appropriate, or by Student’s *t*-test. Significance was set at an alpha of 0.05 and any significant interaction effects were further analyzed by Bonferroni’s post hoc test. 

#### 2.7.2. Proteomics Data

Chromatographic/spectral alignment, feature extraction, data filtering, and statistical analysis was carried out using Nonlinear Dynamics Progenesis LC-MS software (www.nonlinear.com). Raw data files were imported into the program and detected mass spectral features were aligned based on the retention time of the detected *m*/*z* peaks based on a randomly selected reference run. All other runs were automatically aligned to the reference run to minimize retention time variability between runs. No adjustments were necessary in the *m*/*z* dimension, due to the high mass accuracy of the spectrometer (typically <3 ppm). All runs were selected for detection with an automatic detection limit. Features within retention time ranges of 0–5 min were filtered out, as were features with charge state greater than +6 or singly charged peptides (as no MS/MS fragmentations were taken for these charge states during data collection) for reduction of false positive peptide assignments. A normalization factor based on the use of the median and the median absolute deviation was then calculated to account for the approximation of the variance to remove the influence of outliers, and to account for differences in sample load between injections. The experimental design grouped multiple injections from each condition. Stringent conditions were set in in-house MASCOT search engine (Matrix Science, Boston, MA) to filter out low scoring identified peptides by imposing a confidence probability score (*p*) of <0.05. A protein was quantified if it contained at least two unique identified peptides. The filtered MS/MS spectral features along with their precursor spectra were exported in the form of an .mgf file (Mascot generic file) for database searching using the Mascot algorithm [22]. These data were searched against the Uniprot (*Rattus norvegicus*) database. The confidence level was set to 95% within the MASCOT search engine for peptides assigned hits based on randomness. MS/MS analysis was based on the use of trypsin and the following variable modifications: carbamidomethyl (Cys), Oxidation (Met), Phospho (Ser, Thr, Tyr). Other search parameters included peptide mass tolerance of ±15 ppm, fragment mass tolerance of ±0.5 Da, and maximum missed cleavages of 3. A decoy search (based on the reverse sequence search) was performed to estimate the false discovery rate (FDR), with a setting of acceptable protein ID having FDR of 2%. A protein was considered to be positively identified if there were two or more significantly labeled unique peptides (bold red based on Mascot MOWSE scoring). The Mascot significance score match is based on a MOWSE score, and it relies on multiple matches to more than one peptide from the same protein. The Mascot search results were exported to an .xml file using a significance cutoff of <0.05, and ion score cutoff of 28, and a requirement of at least one bold (first time any match to the spectrum has appeared in the report) and red (top scoring peptide match for this spectrum) peptide. The .xml file was then imported into the Progenesis LCMS software, where search hits were assigned to corresponding detected features (post-translational modifications), identified as described above. Verification of phosphorylation site(s) was carried out using the PhosphoRS algorithm [23], and phosphorylated peptides with PhosphoRS probability greater than 0.7 (confidently assigned from the MS/MS fragmentation spectra) were considered in our analyses.

Once proteins and protein modifications for each peptide were determined, the normalized intensity values for each sample were averaged within each group. When the same modification was identified on a protein as separate peptides (due to charge state or cleavage differences), the peptide with the lowest coefficient of variation was chosen as the representative readout for that phosphorylation event. However, in cases where the multiple peptides did not show the same general statistical relationships between groups, then both peptides were kept in the analysis. The four groups were compared by ANOVA to identify *p*-values of potential differences between any of the four groups. The −log10 value of these *p*-values was then plotted against the log2 ratio of the average intensity for the CORT group relative to the water group, and the EtOH group relative to the sucrose group, in volcano plots. These data were also used to determine the significant main effects of relative phosphopeptide abundance. These data were also analyzed by two-way ANOVA to determine whether there were any interactions in phosphopeptide abundance based on adolescent treatment and reinforcer self-administered. Note that phosphopeptide abundance was not normalized to total protein levels, allowing the possibility that apparent differences in phosphorylation are driven by changes in total protein expression. Nevertheless, the data still indicate a change in the amount of signaling related to that phosphorylation event in the amygdala. Given that the purpose of this study was to discover potential new targets for treating stress-associated alcohol drinking, we present data with *p*-values of *p* < 0.05 for main effects (CORT vs H_2_O treatment or sucrose vs EtOH self-administration) and *p* < 0.1 for interaction effects after correcting for multiple comparisons using the Benjamini–Hochberg procedure, assuming a 10% false discovery rate. Significant interactions were further examined using Tukey’s post-hoc test.

Phosphopeptides showing significant interactions were further analyzed using a principal components analysis and a hierarchical clustering analysis to better visualize the relationship of phosphopeptide abundance between groups. The distance between two clusters was calculated using Ward’s method.

## 3. Results

### 3.1. Experiment 1. Analysis of Adolescent CORT Effects on Adult Ethanol Self-Administration and the Amygdala Phosphoproteome

**Figure 1:**
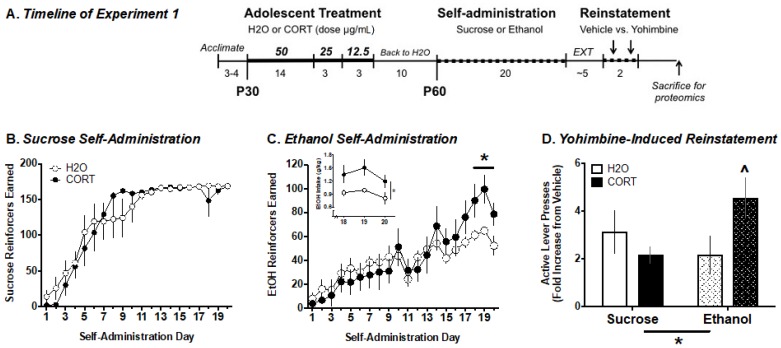
Ethanol and sucrose self-administration and yohimbine-induced reinstatement in H_2_O- and CORT-exposed animals. (**A**) Experimental timeline. CORT exposure did not alter sucrose self-administration (**B**), but increased the response for ethanol during the last three days of self-administration (**C**) and ethanol (g/kg body weight) intake (**C**), inset. Pairwise comparisons following a significant reinforcer × exposure interaction showed a strong trend for increased yohimbine-induced reinstatement of ethanol, but not sucrose seeking in CORT-exposed rats (**D**). Thus, CORT exposure selectively alters ethanol-motivated behavior. Data are presented as the mean ± the standard error of the mean (SEM). * *p* < 0.05’ ^ *p* = 0.06.

A total of 32 rats were exposed to CORT (n = 16) or normal tap water (n = 16) in adolescence. In adulthood, half of the rats were trained to self-administer either sucrose or ethanol + saccharin, with n = 8 in each treatment group (sucrose–H_2_O, sucrose–CORT, EtOH–H_2_O, EtOH–CORT). Figure 1A illustrates the experimental design.

#### 3.1.1. Ethanol Self-Administration

In adulthood, adolescent CORT-exposed and control rats were trained to self-administer ethanol or sucrose. Factorial ANOVA analysis of overall numbers of reinforcers earned, active lever presses, and magazine entries across self-administration sessions as a function of adolescent treatment (H_2_O vs. CORT) and reinforcer type (sucrose vs. EtOH), indicated no treatment × reinforcer interactions (all *p* > 0.05). However, there were significant effects of time and time × reinforcer interactions for reinforcers earned (F_(19, 8)_ = 58.22, *p* < 0.001; F_(19, 8)_ = 43.99, *p* < 0.001), indicating that sucrose self-administration (Figure 1B) was acquired more quickly for both treatment groups. Inspection of the ethanol self-administration data indicated that once animals had acquired stable self-administration (e.g., the final 3 days of testing), there was a separation between the CORT-exposed and control groups in the number of ethanol reinforcers earned (Figure 1C). Analysis of the number of reinforcers earned on the last three days of self-administration revealed a significant reinforcer type by adolescent exposure interaction (F_(1, 26)_ = 8.10, *p* = 0.009), and subsequent analysis by separate two-way ANOVAs for each reinforcer type indicated that there was only a significant effect of adolescent CORT exposure on reinforcers earned in rats responding for ethanol (F_(1, 13)_ = 17.14, *p* < 0.001), but not for sucrose (F_(1, 13)_ = 1.829, *p* > 0.05). Therefore, adolescent CORT exposure resulted in a significant increase in the number of ethanol reinforcers earned once self-administration was acquired, but did not affect self-administration of sucrose (Figure 1B,C). Similarly, analysis of ethanol intake (g/kg) during the same time frame revealed a significant effect of adolescent condition (F_(1, 26)_ = 6.94, *p* = 0.02), but no interaction with session day (F_(2, 26)_ = 0.32, *p* > 0.05), indicating that the adolescent CORT-exposed animals self-administered significantly more ethanol as a g/kg dose during all three of the last self-administration sessions (Figure 1C, inset).

#### 3.1.2. Yohimbine-Induced Reinstatement of EtOH vs. Sucrose Seeking

Following self-administration and subsequent extinction training, rats were tested for yohimbine-induced reinstatement. Data were expressed as a fold change in responses on the active lever following yohimbine relative to vehicle injection (Figure 1D). A two-way ANOVA revealed a significant reinforce type × adolescent exposure interaction (F_(1, 27)_ = 4.9, *p* = 0.036). Subsequent *t*-tests comparing the effects of adolescent exposure revealed a trend (*p* = 0.06) for increased yohimbine-induced reinstatement of ethanol, but not sucrose (*p* = 0.33) seeking. These results indicate that adolescent CORT exposure may enhance sensitivity to the ability of a pharmacological stressor to induce reward seeking, but that this effect is selective for responding for ethanol. 

#### 3.1.3. Phosphoproteomic Analysis

**Figure 2:**
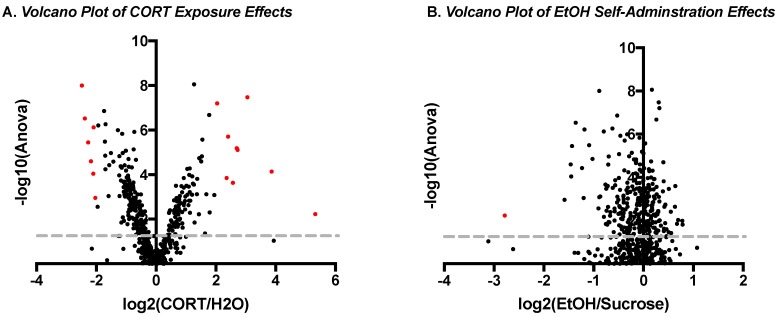
Volcano plots of main effects of adolescent exposure and adult self-administration. Plots are shown comparing main effect of adolescent CORT exposure (**A**) or the main effect of ethanol (EtOH) self-administration experience (**B**). Each plot shows the identified phosphopeptides based on the log2 effect size of adolescent CORT relative to H_2_O exposure on the *x*-axis and the −log10 of the ANOVA *p*-value on the *y*-axis. The gray lines represent the Benjamini–Hochberg-corrected significance point for *p* < 0.05. Points in red represent phosphopeptides that are both highly significantly different and have a large effect size (>4-fold difference).

Two weeks after the reinstatement test, rats were euthanized and their brains were analyzed for changes in the levels of phosphorylated proteins in the amygdala. A discovery-based mass spectrometry approach was used to identify potentially novel biological signaling differences in the brains of rats that self-administered sucrose vs ethanol or that were exposed to CORT in adolescence, and their interaction. A total of 156 unique proteins were identified on which sites of phosphorylation could be resolved. Within these proteins, 478 unique phosphorylation patterns were identified, and of these, 270 phosphopeptides were significantly regulated in at least one of the experimental conditions (Suppl. Table S1, phosphopeptides above yellow row are significant). Next, volcano plots were created to compare the magnitude of change in phosphopeptide abundance based on the main effect of adolescent CORT exposure (Figure 2A) versus the main effect of ethanol self-administration (Figure 2B) relative to the −log10 of the *p*-value from the ANOVA to identify highly significant differences (*y*-axis) of large effect size (*x*-axis). This analysis revealed that adolescent CORT exposure produced 16 changes in protein phosphorylation (red dots in Figure 2A) that were both significantly different from H_2_O exposure (points above gray line = *p* < 0.05 after correcting for multiple comparisons) and that were of large effect size (either increases or decreases with an effect size greater than a four-fold change from H_2_O exposed control = log2(ratio) >2 or <−2). On the other hand, there was only one significant difference of large effect size identified, based on the reinforcer that was previously self-administered (Figure 2B), suggesting that adolescent CORT exposure had a larger long-term effect on the amygdala phosphoproteome than the prior ethanol self-administration experience. Indeed, the protein seemingly regulated by ethanol self-administration, microtubule-associated protein 2 (MAP2), was also regulated by CORT exposure, and both effects were likely driven by a few large values in the CORT–sucrose group.

**Figure 3:**
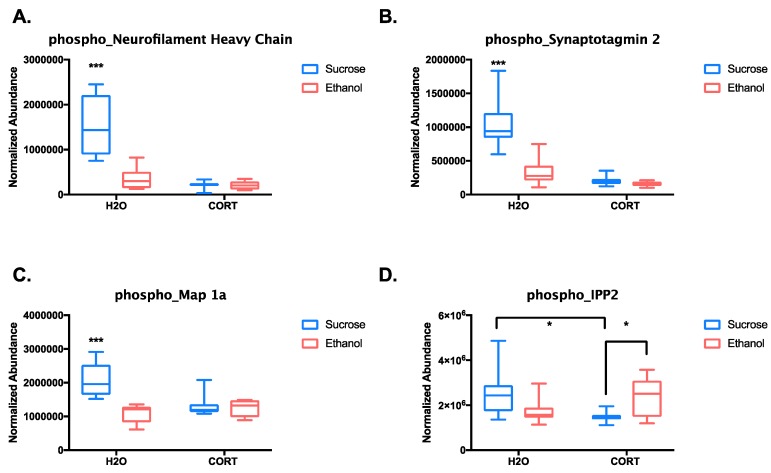
Box plots of phosphopeptides with significant interactions. Plots show the effect of adolescent exposure versus reinforcer self-administered in adulthood for four of the phosphopeptides found to have significant interaction effects after two-way ANOVA: (**A**) neurofilament heavy chain (SPAEAKpSPAEAKPPAEAK), (**B**) synaptotagmin 2 (GGQDDDDAETGLpTEGEGEGEEEKEPENLGK), (**C**) Map 1a (GFKpSPPCEDFSVTGESEK), and (**D**) IPP2 (EQEpSpSGEEDNDLSPEER). Significant interactions were followed by Tukey’s post-hoc test, *** *p* < 0.0001, * *p* < 0.05.

We went on to test for potential interaction effects between adolescent CORT exposure and ethanol self-administration. After correcting for multiple comparisons, significant interactions (*p* < 0.1) were identified for 10 phosphopeptides (Suppl. Table S2). Of these 10, seven were different phosphopeptides from the neurofilament heavy and medium chain proteins. Figure 3A shows the quantitative difference between groups from one of these neurofilament phosphopeptides, which was representative of the pattern of results observed for all of the neurofilament phosphopeptides. Tukey’s post-hoc analysis revealed that phosphorylation of the neurofilament proteins was highest in the adolescent control group that self-administered sucrose (*p* < 0.0001 relative to all other groups). A similar pattern was observed for two of the other phosphopeptides identified, synaptotagmin 2 and Map 1a (all *p* < 0.0001 comparing H_2_O–sucrose to all other groups; Figure 3B,C). Therefore, either prior adolescent CORT exposure or ethanol self-administration resulted in reduced phosphorylation of these peptides relative to controls. The only phosphopeptide to show a different pattern of results was IPP2 (protein phosphatase inhibitor 2, PPP1R2). Phosphorylation of IPP2 on serines 121 and 122 was reduced in the adolescent CORT-exposed rats that self-administered sucrose relative to H_2_O–sucrose controls (*p* = 0.012), but CORT-exposed rats that self-administered ethanol showed a significant reversal of this effect (*p* = 0.035; Figure 3D). Thus, with the exception of IPP2, all significant interactions between adolescent exposure groups and reinforcer types indicated that self-administration of ethanol could reduce protein phosphorylation in the adolescent H_2_O-exposed group to the levels of adolescent CORT-exposed rats, while ethanol produced no further effects beyond the CORT exposure. 

**Figure 4:**
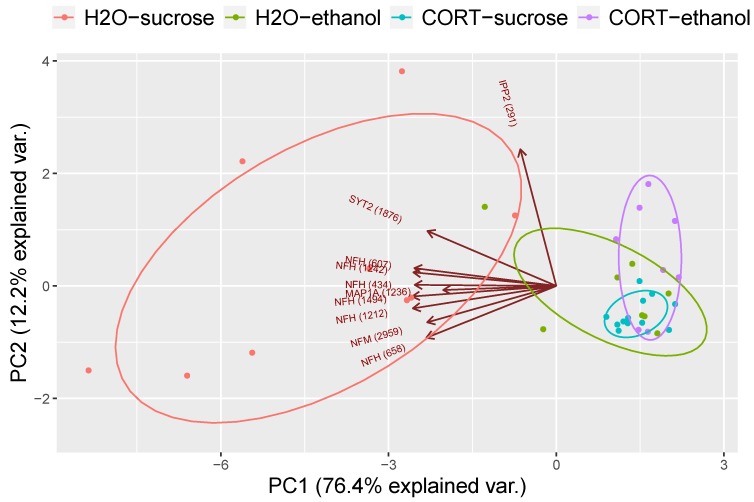
Principal components analysis of significant interactions. Plot shows the first principal component on the *x*-axis and second principal component on the *y*-axis. Each colored ellipsis represents a different group and the clustering of the CORT groups shows that much of the variance between groups could be explained by adolescent CORT exposure. The H_2_O–sucrose group was the most different, suggesting that ethanol self-administration shifted the H_2_O group to be more similar to adolescent CORT group. Overlaid is a biplot (brown circle and arrows) indicating the majority of the phosphopeptides in the H_2_O–sucrose group are in greater abundance than the other three groups.

**Figure 5:**
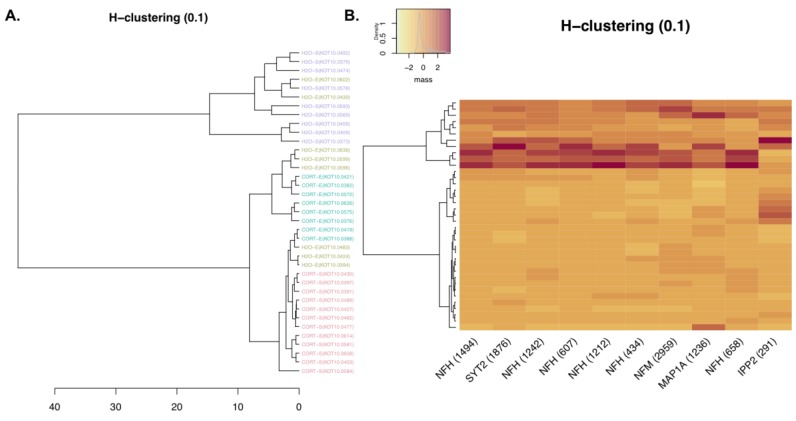
Hierarchical clustering analysis. (**A**) Hierarchical clustering of individual samples based on the abundance of the 10 phosphopeptides significantly different among groups showing general clustering of adolescent H_2_O and CORT groups, with the H_2_O–ethanol group showing mixed clustering between the two. (**B**). Heat map of clusters versus phosphopeptides with darker colors representing greater abundance of the phosphopeptide.

These results were further supported by a principal component analysis (PCA) and a hierarchical clustering analysis. Figure 4 illustrates that the first principal component explained the majority of the variance, with the H_2_O–sucrose group showing a concentration ellipse that did not overlap with the other three groups. Overlaying the PCA plot is a biplot indicating that the H_2_O–sucrose group generally had higher values for each phosphopeptide relative to the other groups, suggestive of reduced phosphorylation in the experimental groups. In addition, hierarchical clustering analysis based on the abundance of the 10 phosphopeptides was significantly different among groups, showing that the adolescent H_2_O- and CORT-exposed groups largely clustered separately, independent of the reinforcer self-administered, with the exception of some of the rats in the H_2_O–ethanol group, which clustered more closely with the CORT groups (Figure 5).

Next, due to the large effect of adolescent CORT exposure on the amygdala phosphoproteome, independent of self-administration condition, we focused our analysis on the 16 significantly regulated phosphopeptides shown in red in Figure 2A. The identity of each of the phosphopeptides is listed in Table 1, where the protein, modified peptide sequence, log2 magnitude of change, and *p*-value from the ANOVA are given. The phosphorylated residues are shown in red.

**Figure 6:**
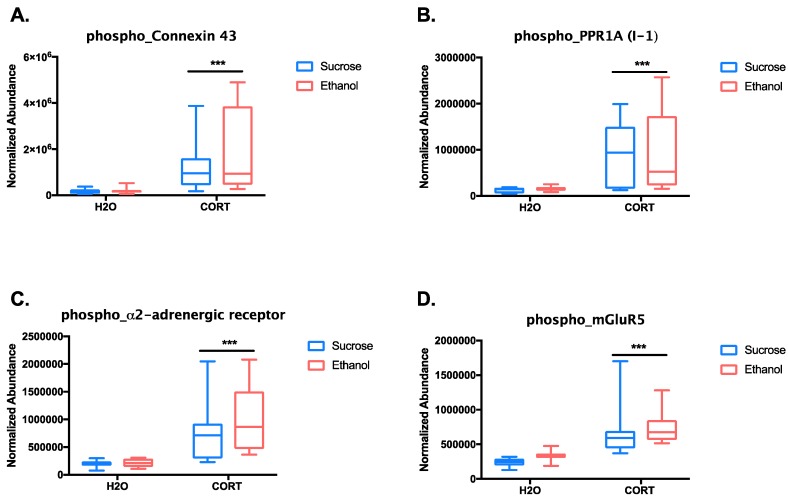
Box plots of phosphopeptides highly differentially regulated by adolescent CORT exposure. Plots show the effect of adolescent exposure group versus reinforcer self-administered in adulthood for four of the phosphopeptides found to have highly significant differences of large effect size based on adolescent CORT exposure with relevance to alcohol use disorders: (**A**) connexin 43 (VAAGHELQPLAIVDQRPSpSRApSpSR), (**B**) PPR1A (RRPTPATLVLTpSDQpSpSPEVDEDRIPNPLLK), (**C**) α_2A_AR (DGDALDLEEpSpSpSpSEHAERPQGPGKPER), and (**D**) mGluR5 (pSPpSPISTLSHLAGSAGR). Significant main effects of CORT, *** *p* < 0.0001.

Overall, adolescent CORT exposure appeared to produce increased phosphorylation of the microtubule-associated protein MAP2, particularly in the N-terminal domain, while the phosphorylation of neurofilament proteins was decreased. These data are suggestive of CORT-induced structural changes in the amygdala, though the exact functions of the phosphorylation sites identified are currently unknown. Of interest for alcohol use and other psychiatric disorders, adolescent CORT exposure also regulated the gap junction protein, connexin43, the protein phosphatase 1 regulatory subunit 1a (PPP1R1a), which is also known as inhibitor 1 (I-1), and the α_2A_AR. In addition, the most highly statistically significant change in phosphopeptide abundance between groups was for the metabotropic glutamate receptor 5 (mGluR5), though the magnitude of effect was slightly less than 4-fold. Given the known relevance of mGluR5, particularly in the amygdala, for alcohol-motivated behaviors [24,25,26,27,28], we further inspected the two-way interaction between adolescent treatment and reinforcer self-administered for this receptor and the other highly regulated phosphopeptides. A Two-way ANOVA revealed the main effects of CORT in increasing the abundance of each of these phosphopeptides (connexin 43: (F_(1, 33)_ = 14.44, *p* < 0.001); I-1: (F_(1, 33)_=15.5, *p* < 0.001); α_2A_AR: (F_(1, 33)_ = 24.17, *p* < 0.001); mGluR5: (F_(1, 33)_=26.64, *p* < 0.001)), with no effect of reinforcer consumed during self-administration (all *p* > 0.25; Figure 6A–D). 

We next determined the potential functional implications of the phosphorylation events observed. The function of the phosphorylation sites on I-1 (Ser43, Ser46, and Ser47) and mGluR5 (Ser1014 and Ser1016) are unknown. On the other hand, increased phosphorylation of connexin43 was found on Ser365, Ser368, and Ser369, which have been described previously [29]. In particular, phosphorylation of Ser368 is known to decrease the permeability of gap junctions, and it is thought to be mediated by protein kinase C (PKC) [30,31]. Thus, adolescent CORT exposure may lead to long-lasting changes in neural signaling via gap junctions in the amygdala.

Finally, increased phosphorylation of the α_2A_AR was found on four consecutive serines (366–369), which are a known substrate of the G-protein coupled receptor kinase 2 (GRK2) [19,32]. Phosphorylation at these sites mediates agonist-stimulated receptor desensitization, association with arrestin, decoupling from the G-protein, and clathrin-mediated endocytosis [19,32]. Thus, we predicted that the adolescent CORT-treated rats would have a reduced sensitivity to α_2A_R-mediated signaling, which could result in an increase in norepinephrine release and post-synaptic signaling in the brain, as the normal autoreceptor-mediated brake on noradrenergic-transmission would be impaired. Given the large literature on the involvement of heightened noradrenergic signaling, particularly in the amygdala, for both stress- and alcohol-related behaviors, including the potential clinical use of α_2A_AR agonists as a treatment for alcohol use disorders [33,34,35], GRK2-mediated phosphorylation of α_2A_AR after adolescent CORT exposure is a strong candidate as a mediator of increased motivation for alcohol. Therefore, we decided to directly test if inhibition of GRK2 in the amygdala could reduce alcohol-motivated behaviors in adolescent CORT- or H_2_O-exposed rats.

### 3.2. Experiment 2. Determining the Role of GRK2 in Regulating Ethanol Motivated Behaviors

**Figure 7:**
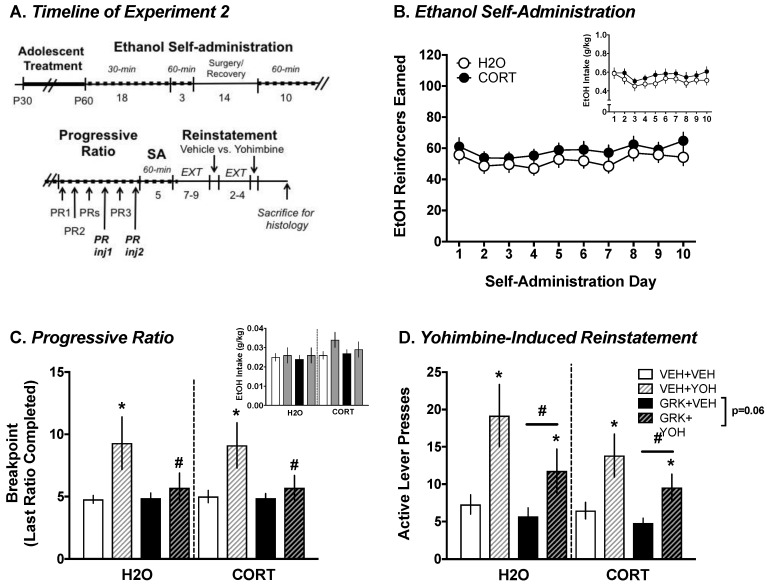
Inhibition of GRK2 attenuates yohimbine-induced increases in ethanol-motivated behavior. (**A**) Experimental timeline. (**B**) CORT exposure consistently, but not significantly, tended to increase reinforcers earned and intake (inset) during ethanol self-administration. (**C**) Intra-basolateral amygdala inhibition of GRK2 blocks yohimbine-induced increases in breakpoint during progressive ratio testing, though the overall low number of reinforcers earned precluded the detection of differences in ethanol intake (inset). (**D**) GRK2 inhibition significantly reduced the reinstatement of ethanol seeking and tended to reduce the effects of yohimbine in increasing reinstatement. * *p* < 0.05 yohimbine (YOH) vs. vehicle (VEH); # *p* < 0.05 GRK2i vs. vehicle.

A total of 94 rats were exposed to CORT (n = 46) or normal tap H_2_O (n = 48) in adolescence at the University of Pittsburgh as in Experiment 1. Of these rats, 26 were ultimately excluded from data analysis due to failure to meet acquisition criteria, misplaced cannula, or death during surgery. Thus, the final sample sizes for this experiment were n = 34 for both CORT and H_2_O groups (n = 16–17 each for GRK2i- and vehicle-treated rats). Figure 7A illustrates the experimental design.

#### 3.2.1. Ethanol Self-Administration

To more closely equate the results of Experiment 2 to those of Experiment 1, analyses were conducted on the 10-day postoperative self-administration period, during which time rats were tested in 60 min sessions. Mixed factorial ANOVAs with adolescent exposure (CORT vs. H_2_O) as the between-subjects factor and day (10) as the within-subject factor revealed significant main effects of day for reinforcers earned ((F_(9, 702)_ = 3.208, *p* < 0.001); Figure 7B) and ethanol intake (g/kg; (F_(9, 702)_ = 3.18, *p* = 0.001); (Figure 7B, inset); however, day-to-day variability was not systematic. Similar patterns evident for all other self-administration parameters (e.g., active lever presses, magazine entries; data not shown). Though no statistically significant main effects of or interactions involving adolescent exposure were found for any outcome measure, CORT-exposed rats consistently showed greater ethanol-motivated behavior compared to H_2_O-exposed controls.

#### 3.2.2. Progressive Ratio Testing: Yohimbine vs. Vehicle 

Mixed factorial ANOVAs with adolescent group (CORT vs. H_2_O) and infusion (GRK2i vs. vehicle) as the between-subjects factors and injection (yohimbine vs vehicle) as the within-subject factor revealed an overall main effect of injection (F_(1, 64)_ = 10.997, *p* = 0.002), and an injection × infusion interaction (F_(1, 64)_ = 5.085, *p* = 0.028) for breakpoint (last ratio completed), with the yohimbine-injected rats showing greater “willingness to work” for ethanol than vehicle-injected rats, and a GRK2i-mediated reduction in these measures was only observed in yohimbine-injected rats (t(66) = 2.229, *p* = 0.029); Figure 7C. Similar patterns were evident for other self-administration parameters (e.g., reinforcers earned, active lever presses; data not shown). Though the analysis of ethanol intake did not reveal significant effects of injection or infusion, rats only earned roughly 3–4 reinforcers on average during PR testing, thus making it difficult to detect differences in very low levels of intake (Figure 7C, inset). No main effect of adolescent group was evident during PR. 

#### 3.2.3. Yohimbine-Induced Reinstatement

Mixed factorial ANOVAs with adolescent exposure (CORT vs. H_2_O) and infusion (GRK2i vs. vehicle) as the between-subjects factors and injection (yohimbine vs. vehicle) as the within-subject factor revealed significant main effects of injection (F_(1, 63)_ = 26.169, *p* < 0.001) and infusion (F_(1, 63)_ = 4.293, *p* = 0.042) for active lever presses during reinstatement (Figure 7D). Yohimbine-injected rats responded more than vehicle-injected rats, and GRK2i-infused rats responded less on the active lever than rats infused with the GRK2i vehicle. An exploratory analysis indicated that like during PR testing, yohimbine-induced increases in ethanol-motivated behavior tended to be attenuated (*p* = 0.06) in GRK2i-infused rats relative to those that received vehicle infusion. No main effect of the adolescent group was evident during reinstatement. Taken together, these results indicate that intra-BLA inhibition of GRK2 reduces yohimbine-induced increases in ethanol-motivated behavior.

## 4. Discussion

In the present series of studies, we first examined the impact of chronic exposure to the glucocorticoid stress hormone corticosterone (CORT) during adolescence (PND 30–50) on ethanol-motivated behaviors and on the amygdala phosphoproteome (Experiment 1). We found that rats chronically exposed to CORT during adolescence self-administered significantly more of a sweetened ethanol solution than control rats, once self-administration was acquired. Further, CORT-exposed rats displayed enhanced yohimbine stress-induced reinstatement in ethanol-reinforced, but not sucrose-reinforced rats. Importantly, chronic CORT exposure increased phosphorylation of a series of serine residues in the α_2A_ adrenergic receptor protein in the amygdala, at which yohimbine exerts its pharmacological action. We then targeted the kinase that phosphorylates these residues, G protein-coupled receptor kinase 2 (GRK2), in experiments aimed at determining whether GRK2 inhibition would alter ethanol-motivated behaviors as a function of chronic (CORT exposure during adolescence) and/or acute (injection of yohimbine) stress exposure (Experiment 2). While we only uncovered statistical trends for the ability of chronic adolescent CORT exposure to increase ethanol self-administration in Experiment 2, we found that inhibition of GRK2 in the BLA significantly attenuated yohimbine-induced increases in ethanol-motivated behavior, regardless of adolescent experience. These findings suggest that GRK2 inhibition is a promising target for reducing stress-induced increases in ethanol-motivated behaviors. 

Prolonged stress exposure during adolescence has been shown to increase the vulnerability of developing psychiatric disorders, including alcoholism later in life [5,6]. Prior studies have shown that chronic CORT exposure in adolescence subsequently increases impulsivity [10] in adulthood. This indicates that there are long-lasting effects of elevated glucocorticoid levels during adolescence that could increase the risk of maladaptive behavior. However, while some studies have shown that post-weaning isolation stress increases ethanol-motivated behaviors [12,14,36], the present and previous studies utilizing the chronic CORT model in adolescence [15] showed inconsistent effects in the ability of CORT to significantly augment ethanol-motivated behaviors. The significant increase in ethanol self-administration during the final three days of training in Experiment 1 is paralleled by consistent trends for prior CORT exposure to augment responses for ethanol in Experiment 2, and cue-induced reinstatement in female rats [15]. This disparity could be due to environmental differences in the two facilities in which the present experiments were conducted. Indeed, large differences in behavioral outcomes have been documented, even when experimental conditions (other than facility) are held constant [37,38]. It is possible that these unavoidable changes in husbandry could have resulted in the diminished CORT effects on drinking in Experiment 2, potentially leading to a decreased sensitivity to stress. For example, other studies have identified differences in ingestive behavior [39] and stress/anxiety-related responses [40] in rodents housed in open-style (like Experiment 1) versus individually ventilated (like Experiment 2) cages, potentially leading to the lack of a robust effect of CORT on subsequent ethanol intake in the latter experiment. Our laboratory has conducted experiments to directly determine if light cycle phase or degree of food restriction during ethanol self-administration was responsible for the differential effects of adolescent CORT exposure between Experiments 1 and 2, and neither of these factors was found to consistently influence our results. Future studies could test exposures to higher concentrations of CORT to potentially overcome any stress-buffering effects that the current facility may have, to improve the replicability of these results. 

Regardless of the sensitivity of prior chronic CORT exposure in adolescence to alter subsequent ethanol self-administration, the proteomics analysis identified a number of differentially phosphorylated proteins in the amygdala of the CORT-exposed rats that did show greater ethanol self-administration in Experiment 1, and these could potentially be targeted to treat alcohol use disorders. The effect of adolescent CORT exposure was greater than ethanol self-administration experience alone, suggesting that elevated glucocorticoids in adolescence may produce a long-lasting vulnerability that is not substantially exacerbated by ethanol intake. In addition, while there were a few phosphopeptides that exhibited significant interactions between adolescent treatment and the reinforcer that was self-administered, almost all of these phosphopeptides were of the highest abundance in the H_2_O–sucrose group, with ethanol self-administration bringing the abundance in the H_2_O group down to the level of the CORT exposed rats. These data are intriguing and suggestive that three weeks of 1 hour daily ethanol exposure may shift the molecular activity of the amygdala of control rats to a state that is more similar to rats that were exposed to chronic CORT in adolescence. 

Importantly, the majority of significantly regulated phosphopeptides were observed in the adolescent CORT group independent of the reinforcer self-administered in adulthood, again suggesting that adolescent CORT exposure produces profound effects on the amygdala phosphoproteome, including proteins that are associated with alcohol use disorders, which may indicate heightened vulnerability to the effects of alcohol. In particular, increased phosphorylation of four serine residues in the third intracellular loop of the α2AR, which are a GRK2 substrate, was of particular interest, due to evidence pointing to the potent role of adrenergic signaling, particularly in the amygdala, in ethanol drinking and seeking. It has long been recognized that the noradrenergic system plays a critical role in the development of alcohol use disorders, but only recently has interest been revitalized in targeting this system with respect to AUD treatment [34]. Preclinical studies have shown that downregulation of noradrenergic signaling, via α_2A_R agonism (clonidine; [35]), or antagonism of α1AR (prazosin; [41]) or βAR (propranolol; [42]), reduces ethanol drinking and seeking in high-consuming animals. Similar treatment approaches have been undertaken to treat comorbid post-traumatic stress disorder (PTSD) and AUD [33,43], as these disorders frequently co-occur, lead to significant disability, and are difficult to treat effectively [44].

Further, early life stress reduces norepinephrine in the amygdala [45] and α_2A_R (*adra2a*) gene expression in the hypothalamus [46], which leads to increased anxiety-like behavior and ethanol drinking, respectively, and enhances ethanol-induced norepinephrine levels in the BLA [47]. Taken together, these studies suggest that early life stress alters noradrenergic functioning, consistent with the enhanced phosphorylation, and likely subsequent internalization of the autoreceptor α_2A_R in CORT-exposed rats. Moreover, ethanol exposure can lead to a heightened noradrenergic response, consistent with the enhanced yohimbine-related ethanol-motivated behavior that was mitigated by blocking GRK2-induced α_2A_R phosphorylation in both H_2_O- and CORT-exposed rats. Our results add to a small, but growing number of studies examining the role of GRK-mediated phosphorylation of metabotropic receptors, such as serotonin 5-HT2A [48], dopamine D1/D2 [49,50], cannabinoid [51], and mu opioid receptors [52], in models of substance use disorders, and suggest that GRKs should be further studied as potential targets for novel treatment development. Finally, the proteomics results also identified novel potential targets for treating alcohol use disorders, such as gap junction signaling through connexin43, and provide further support for the development of mGluR5 modulators as treatments for substance use and other disorders [25].

## 5. Conclusions

The present studies expand on previous findings of enhanced vulnerability to maladaptive behavior following exposure to persistently elevated glucocorticoid levels by demonstrating that chronic adolescent CORT exposure can lead to heightened ethanol drinking, enhancement of yohimbine stress-induced ethanol seeking, and increased phosphorylation of α2ARs at residues that are the substrates for GRK2-mediated receptor desensitization/internalization. We then showed that blocking these reductions in α2AR function by inhibiting GRK in the BLA blocks yohimbine stress-induced ethanol seeking, regardless of prior CORT exposure. These results suggest that altering GRK activity, and/or facilitating noradrenergic autoinhibition, are promising targets for reducing stress-related alcohol use.

## Figures and Tables

**Table 1:** Phosphopeptides significantly regulated by adolescent CORT exposure.

Top Up-Regulated Phosphopeptides			
Protein	Peptide Sequence + Phosphorylation Sites (in Red)	Log2(CORT/H_2_O)-Magnitude	*p*-Value (ANOVA)
Microtubule-associated protein 2	RLSNVSSSGSINLLESPQLATLAEDVTAALAK	5.325915864	0.005821436
Microtubule-associated protein 2	RLSNVSSSGSINLLESPQLATLAEDVTAALAK	3.863577679	7.21 × 10^−5^
Gap junction alpha-1 protein	VAAGHELQPLAIVDQRPSSRASSR	3.054725626	3.34 × 10^−8^
Microtubule-associated protein 2	RLSNVSSSGSINLLESPQLATLAEDVTAALAK	2.726525438	7.69 × 10^−6^
Protein phosphatase 1 regulatory subunit 1A	RRPTPATLVLTSDQSSPEVDEDRIPNPLLK	2.69773257	6.46 × 10^−6^
Canalicular multispecific organic anion transporter 2	IPLNLLPQLISGMTQTSVSLK	2.568107399	0.000230896
Microtubule-associated protein tau	HLSNVSSTGSIDMVDSPQLATLADEVSASLAK	2.409694538	1.97 × 10^−6^
Microtubule-associated protein 2	RLSNVSSSGSINLLESPQLATLAEDVTAALAK	2.359792109	0.000139602
Alpha-2A adrenergic receptor	DGDALDLEESSSSEHAERPQGPGKPER	2.043347801	6.23 × 10^−8^
**Top Down-Regulated Phosphopeptides**			
Neurofilament light polypeptide	AEEAKDEPPSEGEAEEEEK	−2.48762892	9.91 × 10^−9^
Neurofilament heavy polypeptide	TLDVKSPEAK	−2.38663396	2.96 × 10^−7^
Neurofilament heavy polypeptide	SLAEAKSPEK	−2.276582671	3.53 × 10^−6^
Neurofilament heavy polypeptide	SPAEAKSPAEAKPPAEAK	−2.178346111	2.49 × 10^−5^
Neurofilament heavy polypeptide	SPVEVKSPEK	−2.100576776	9.08 × 10^−5^
Neurofilament heavy polypeptide	SPAEAKSPAEVK	−2.093897637	7.44 × 10^−7^
Neurofilament medium polypeptide	AEEEGGSEEEVGDKSPQESK	−2.035085213	0.001096364

## Supplementary Materials

The following are available online at http://www.mdpi.com/2227-7382/6/4/41/s1. Table S1: Protein List, Table S2: Two-way ANOVA Interaction Table.
